# Pazopanib may reduce bleeding in hereditary hemorrhagic telangiectasia

**DOI:** 10.1007/s10456-018-9646-1

**Published:** 2018-09-06

**Authors:** Marie E. Faughnan, James R. Gossage, Murali M. Chakinala, S. Paul Oh, Raj Kasthuri, Christopher C. W. Hughes, Justin P. McWilliams, Joseph G. Parambil, Nicholas Vozoris, Jill Donaldson, Gitanjali Paul, Pamela Berry, Dennis L. Sprecher

**Affiliations:** 1 Toronto HHT Program, Division of Respirology, Department of Medicine, St. Michael’s Hospital, University of Toronto, Toronto, ON Canada; 2 Li Ka Shing Knowledge Institute of St. Michaels Hospital, 30 Bond St, Toronto, ON M5B-1W8 Canada; 3 Division of Pulmonary and Critical Care Medicine, Augusta University, Augusta, GA USA; 4 Division of Pulmonary and Critical Care Medicine, Washington University, St. Louis, MO USA; 5 Barrow Aneurysm & AVM Research Center, Barrow Neurological Institute/Dignity Health, Phoenix, AZ USA; 6 Division of Hematology and Oncology, Department of Medicine, UNC School of Medicine, Chapel Hill, NC USA; 7 Department of Molecular Biology & Biochemistry, and Department of Biomedical Engineering, University of California Irvine, Irvine, CA USA; 8 Division of Interventional Radiology, Department of Radiology, David Geffen School of Medicine at UCLA, Los Angeles, CA USA; 9 Cleveland Clinic, Respiratory Institute, Cleveland, OH USA; 10 GlaxoSmithKline, King of Prussia, PA USA; 11 Patient Reported Outcomes, Janssen Global Services, LLC, Horsham, PA USA

**Keywords:** Telangiectasia, Hereditary, Epistaxis, Anemia, Tyrosine kinase inhibitor

## Abstract

Pazopanib (Votrient) is an orally administered tyrosine kinase inhibitor that blocks VEGF receptors potentially serving as anti-angiogenic treatment for hereditary hemorrhagic telangiectasia (HHT). We report a prospective, multi-center, open-label, dose-escalating study [50 mg, 100 mg, 200 mg, and 400 mg], designed as a proof-of-concept study to demonstrate efficacy of pazopanib on HHT-related bleeding, and to measure safety. Patients, recruited at 5 HHT Centers, required ≥ 2 Curacao criteria AND [anemia OR severe epistaxis with iron deficiency]. Co-primary outcomes, hemoglobin (Hgb) and epistaxis severity, were measured during and after treatment, and compared to baseline. Safety monitoring occurred every 1.5 weeks. Seven patients were treated with 50 mg pazopanib daily. Six/seven showed at least 50% decrease in epistaxis duration relative to baseline at some point during study; 3 showed at least 50% decrease in duration during Weeks 11 and 12. Six patients showed a decrease in ESS of > 0.71 (MID) relative to baseline at some point during study; 3/6 showed a sustained improvement. Four patients showed > 2 gm improvement in Hgb relative to baseline at one or more points during study. Health-related QOL scores improved on all SF-36 domains at Week 6 and/or Week 12, except general health (unchanged). There were 19 adverse events (AE) including one severe AE (elevated LFTs, withdrawn from dosing at 43 days); with no serious AE. In conclusion, we observed an improvement in Hgb and/or epistaxis in all treated patients. This occurred at a dose much lower than typically used for oncologic indications, with no serious AE. Further studies of pazopanib efficacy are warranted.

**Electronic supplementary material:**

The online version of this article (10.1007/s10456-018-9646-1) contains supplementary material, which is available to authorized users.

## Introduction

Hereditary hemorrhagic telangiectasia (HHT) is a common genetic disorder affecting between 1 in 5000 to 1 in 10,000 [1] and is characterized by the development of vascular malformations (VMs) and bleeding. Causative mutations in one of several genes, most commonly endoglin (*ENG*) or activin receptor-like kinase 1 (*ACVRL1*) and less commonly *SMAD4*, lead to the characteristic HHT lesions—large vessel arteriovenous malformations (AVMs) and/or small vessel telangiectases [2–4]. Telangiectases on the nasal mucosa lead to recurrent and spontaneous epistaxis, starting as early as childhood. Similarly, telangiectases develop in the gastric and small intestinal mucosa and lead to chronic gastrointestinal (GI) bleeding, typically in adults [5]. Chronic HHT-related bleeding, from either or both nasal and GI sources, causes morbidity in HHT, including anemia and its complications, as well as reduced quality of life (QOL) [6–9].

Traditional, medical, endoscopic, and surgical interventions for epistaxis and GI bleeding in HHT have provided at most short-term success, with concern about cumulative adverse effects and complications with repeated interventions [10–16]. Recently, there is considerable interest in pursuing therapies that target pathogenic pathways in HHT. Several lines of evidence suggest that a loss of *ACVRL1* or *ENG* promotes a pro-angiogenic milieu. Serum and plasma levels of vascular endothelial growth factor (VEGF) are increased in HHT patients [17], and adult-onset AVMs in the brain and skin have been shown to require angiogenic stimulators such as VEGF in addition to ACVRL1 or ENG-deficiency [18, 19]. VEGF neutralizing antibodies have been found to inhibit wound-induced adult-onset AVMs in animal models [19, 20] and so anti-angiogenic therapies are being explored in HHT. Bevacizumab [Avastin], an approved VEGF-A monoclonal antibody used for various solid organ malignancies, has been administered to HHT patients in small case series. The best results in HHT have been reported with intravenous delivery, for symptomatic liver VMs [21] and chronic severe HHT-related bleeding [22]. Variable results have been obtained with topical use for epistaxis [10, 23, 24]. The requirement for parenteral administration, uncertainty over effective dosing, limited durability of effect necessitating repetitive doses, and potential serious side effects have limited the use of bevacizumab [10, 23, 25].

Pazopanib (Votrient) is an orally administered tyrosine kinase inhibitor (TKI) targeting multiple receptor tyrosine kinases (RTKs), and is approved for treating advanced/metastatic renal cell carcinoma and advanced soft tissue sarcomas. Pazopanib blocks VEGF, PDGF, and c-kit receptors. We previously demonstrated that pre-treatment with a chemical equivalent to pazopanib [26] inhibited development of AVMs in the *ACVRL1* knockout mouse.

This prospective, multi-center, dose-escalating study was designed as a proof-of-concept study to demonstrate efficacy of pazopanib on HHT-related bleeding, and also to measure safety in this population. We chose to study patients with chronic HHT-related bleeding, given its significant morbidity and the inadequacy of current therapies. Also, we speculated that the smallest VMs in HHT, such as those leading to nasal and GI bleeding, would be most responsive to anti-angiogenic therapy. We chose to study epistaxis and anemia as co-primary outcomes, also collecting comprehensive data regarding anemia management and QOL.

## Methods

### Study design and oversight

This was designed as an open-label, dose-escalation study of up to four serially performed cohorts of approximately six to eight patients each. Each cohort was to receive progressively higher doses than the prior cohort [50 mg, 100 mg, 200 mg, and 400 mg]. Dose escalation would not occur if the predefined safety stopping criteria were met or at least four patients in a cohort demonstrated efficacy (as measured by epistaxis, hemoglobin (Hgb), transfusion, or iron infusion requirements). Given prior 30 mg dosing in healthy volunteers without toxicity, preclinical evidence of efficacy at exposures equivalent to < 30 mg dosing, and the challenge of elucidating a window between toxicity and efficacy, we initiated dosing at a low-dose of 50 mg [1/16th of the oncologic dose of 800 mg daily]. As noted below, efficacy was achieved at the initial 50 mg dose, thereby terminating the study as per protocol, without any dose escalation. Planned additional study was curtailed due to the sale of the product as part of an oncology portfolio purchase. Results are thus only available for this first cohort of patients.

The study was constructed with a 4–8 weeks run-in period to establish a baseline, followed by 12 weeks of therapy and up to 4 months of follow-up, depending on the success of the first 3 months plus demonstration of continued benefit. Follow-up was therefore decided monthly after drug discontinuation to examine whether further visits were warranted.

The study was funded by Glaxo-Smith-Kline, with a contribution from Novartis to the close-out costs. Sponsor oversight was performed by GSK initially and Novartis during the termination phase. The study design committee included investigators and HHT clinical experts (M.E.F., J.R.G., M.M.C., P.O., R.K., and D.L.S) as well as a representative of the patient advocacy group, Cure HHT. Investigators were trained to conduct the study in accordance with GCPs and the study protocol, as defined in ICH E3, Sect. 9.6 as well as ethical principles that are outlined in the Declaration of Helsinki 2008. An investigator meeting was held, and the study was initiated in 2014. All sites received approval from their local institutional review boards and all patients provided written informed consent. The full details of the protocol are publically available at https://www.gsk-clinicalstudyregister.com/study/201128?search=study&search_terms=201128#fp.

The trial was registered with clinicaltrials.gov (NCT02204371).

### Patient selection

Patients were recruited at five participating HHT Centers of Excellence (designated by Cure HHT). Eligible patients were between 18 and 75 years of age and had possible or definite clinical diagnosis of HHT [27]. In addition, eligible patients had to meet one of the following criteria (A, B OR C):

**A**: Severe epistaxis over the previous 4 weeks defined as an average of at least three episodes of epistaxis per week

- AND a total duration of greater than 15 min per week
- AND requiring iron therapy (oral and/or intravenous)

**B**: Anemia* (Hgb < 11 g/dL) despite receiving iron infusions (≥ 0.5 g elemental iron/month)

- AND iron deficiency (pre-infusion ferritin < 60 ng/mL or transferrin saturation < 20%)
- AND substantial compromise in QOL according to the PI (e.g. lethargic, cannot maintain job, listless, fatigued)

**C**: Anemia* (Hgb < 10 g/dL)

- AND requiring blood transfusions (≥ 2 units/month)

Patients were excluded if current or chronic history of non-HHT liver disease or biliary abnormalities. HHT-related exclusion criteria were (1) untreated cerebral vascular malformations (CVMs); (2) untreated pulmonary AVMs with feeding artery diameter ≥ 3 mm; and (3) symptomatic liver VMs (chronic right upper quadrant pain, symptomatic portal hypertension or heart failure). Other exclusion criteria included non-HHT bleeding disorders, use of anti-angiogenic medication in the past 3 months, and patients with known non-HHT cardiovascular disease.

A patient could withdraw from study treatment at any time at his/her own request, or could be withdrawn at any time at the discretion of the investigator for safety, behavioral or administrative reasons.

### Treatment administration

Pazopanib was provided as 50 mg white to off-white round convex tablets (6.35 mm). Patients were instructed to take 1 tablet whole with water at least 1 h before or 2 h after a meal, once daily.

The FDA mandated at least 6 participants to have completed a set dose prior to advancing to the next dose cohort, based on safety and efficacy. The intent was to minimize toxicity for any benefit.

### Safety monitoring

Bloodwork for liver function tests was collected every 1.5 weeks. Each patient was provided an electronic device along with a blood pressure (BP) cuff to measure BP three times per day, during the last week of the run-in period, throughout the treatment period and during the first week of follow-up.

If ALT > 3x ULN, elevated systolic BP produced symptoms, or systolic BP > 160 mmHg, the test drug was to be discontinued. For milder elevations in BP, an algorithm was followed in order to initiate treatment or intensify ongoing therapy.

### Outcomes measurement

Blood draws occurred every 1.5 weeks [Hgb, ferritin]; pazopanib trough levels were performed every 3 weeks. While lab samples were drawn both at scheduled and pre-infusion visits these latter blood draws were typically performed at an unscheduled visit. Laboratory measures were performed at a central site [Quest], and followed their published guidelines for these standard assays.

Epistaxis (frequency, severity, and duration) was assessed pre-, peri-, and post-study with a daily epistaxis diary. Average epistaxis severity over the past 1 month was assessed using the Epistaxis Severity Score (ESS) measure [28] at Day 1, Week 6, Week 12, and during follow-up. Health-related QOL was assessed using the Medical Outcomes Study 36-item Short Form (SF-36 v2) at Day 1 pre-dose, Week 6, and Week 12. Exit interviews were conducted at the end of treatment (Week 12) or early withdrawal visit.

### Efficacy criteria

The efficacy measures defined for dose progression between cohorts also served as criteria for clinical significance, and comprised change from baseline in the average of three consecutive measures in Hgb, change from baseline in total iron intake over 4 weeks of treatment and change from baseline in duration, frequency and intensity (gushing to non-gushing) of epistaxis over two consecutive weeks. These were computed and are presented qualitatively in Table 1.

**Table 1:**
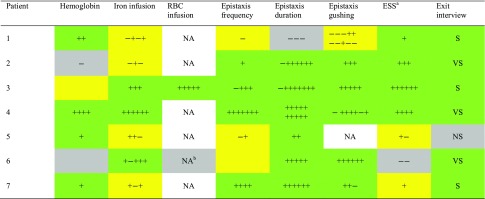
Primary and secondary outcomes

The number of + or − in each box represents the number and order of time periods (both during and after treatment) for which the patient met efficacy criteria for improvement or worsening, respectively, in the parameter as defined in the Methods section. The color of each box is a composite indicator of the perceived overall direction of change during the study based on the pattern and amplitude of the changes: green = overall improved; yellow indicates no overall change; gray indicates overall worse. For example, Patient 1 received 300 mg of iron at baseline and had two occasions where she received 600 mg in one period followed by 0 mg in the following period, suggesting a pattern of no overall change—thus the yellow box color

*ESS* epistaxis severity score, *NA* not applicable as the patient had either zero RBC or zero gushing during baseline period, *NS* not satisfied with treatment, *S* satisfied with treatment, *VS* very satisfied with treatment

^a^ESS was assessed twice during treatment and four times after treatment except in Patient 5 (once during and once after treatment due to early exit from study) and Patient 7 (twice during and twice after treatment)

^b^Patient 6 received no RBC at baseline and two RBC during follow-up and was therefore considered overall worse—thus the gray box color

#### Baseline assessments

Baseline Hgb and ferritin were defined as the average of the last two measurements during the run-in period. Baseline epistaxis frequency, duration, and intensity were calculated as the aggregate of the daily epistaxis diary entries over the last 14 days during the run-in period. Baseline for all other parameters including ESS, QOL, and safety parameters (e.g., vital signs, safety labs, ECGs) was defined as the Day 1 pre-dose measurement.

#### Criteria for clinical significance

It was anticipated that the last weeks of treatment would be most relevant for observing a treatment benefit and therefore data were generally aggregated over 2 or 4 weeks time periods counting back from the end of the treatment period. Since the follow-up period was predominantly targeting relapse, post-treatment data were aggregated in 2–4 weeks periods counting forward from the time of drug discontinuation.

The following changes were considered clinically significant and are the basis for reporting in Table 1. The changes detailed below represent improvements and are notated in Table 1 as “+”. Changes in the opposite direction but of a similar magnitude are notated in Table 1 as “−”. The analyses supporting Table 1 were generated post-hoc.

1. Hemoglobin
  - > 2 gm increase in the average of any three consecutive measures during treatment or after treatment versus baseline: notation +.
2. Iron and red blood cell (RBC) infusion
  - > 50% decrease in any 4 week block versus baseline: notation +.
3. Epistaxis frequency, duration, and gushing
  - ≥ 50% decrease in any 2 weeks block versus baseline: notation +.
4. Epistaxis severity score
  - ESS decrease > 0.71 (MID) at any time point versus baseline: notation +.
5. Exit interview satisfaction:
  - Very satisfied with treatment at exit interview: notation VS.
  - Satisfied with treatment at exit interview: notation S.
  - Not satisfied with treatment at exit interview: notation NS.

### Pharmacokinetics

Pharmacokinetic samples were collected at time-points as detailed above. Centrifuge-acquired plasma from these samples was shipped frozen on dry ice to: Quest Diagnostics Clinical Trials 27027 Tourney Road, Ste 2E Valencia CA 91355 USA. These were subsequently analyzed for pazopanib by PPD, Middleton, WI.

## Results

Between February 25, 2015 and October 19, 2015, nine patients were screened for enrollment into this study. Two patients were screen failures, leaving seven patients for primary analysis. Baseline characteristics of the seven patients are shown in Table 2. All patients had definite HHT as defined by the Curacao criteria and had pathogenic mutations in HHT genes. Six patients received 50 mg daily of pazopanib for the planned 12-week treatment phase (exposure to study drug ranged between 83 and 89 days). One patient (Patient 5) experienced an increase in LFTs and was withdrawn from the dosing phase of the study at 43 days. Compliance with study drug was recorded as 80–120% at all visits.

**Table 2:** Patient demographics and baseline characteristics

Patient	Sex	Age (years)	BMI (kg/m^2^)	Smoker (N = never, F = former)	HHT gene mutated	Baseline ESS	Baseline Hgb (g/dL)	HHT GI bleeding (Y/N)
1	M	58	32	F	*ENG*	4.17	12.2	Y
2	F	43	31	N	*SMAD4*	5.25	10.4	Y
3	F	65	21	F	*ENG*	6.59	7.3	Y
4	M	69	36	N	*ACVRL1*	4.17	9.6	Y
5	M	52	25	N	*ENG*	2.75	11.2	Y
6	F	51	43	N	*ACVRL1*	4.35	11.6	N
7	F	63	25	N	*ACVRL1*	4.06	8.7	Y
Summary/mean	4F/3M	57 (SD = 9)	30 (SD = 7)	5N/2F	3*ACVRL1*/3*ENG*/1*SMAD4*	4.47 (SD = 1.18)	10.1 (SD = 1.7)	6Y/1N

HHT genes mutated are endoglin (*ENG*), activin receptor-like kinase 1 (*ACVRL1*) or *SMAD4*. Baseline ESS is epistaxis severity score (ESS) on Day 1 of dosing. Baseline Hgb is the mean of last two Hgb measures (includes Day1) of the run-in period. HHT GI bleeding is reported Y if patient diagnosed with chronic HHT-related GI bleeding; N if not. Race was white/Caucasian/European heritage for all seven, and non-hispanic ethnicity for all seven

### Primary outcomes

Patients were assessed for efficacy based on the co-primary outcomes of epistaxis severity and hematologic parameters. All patients met one or more pre-defined criteria for efficacy (Table 1).

#### Epistaxis severity

**Fig. 1:**
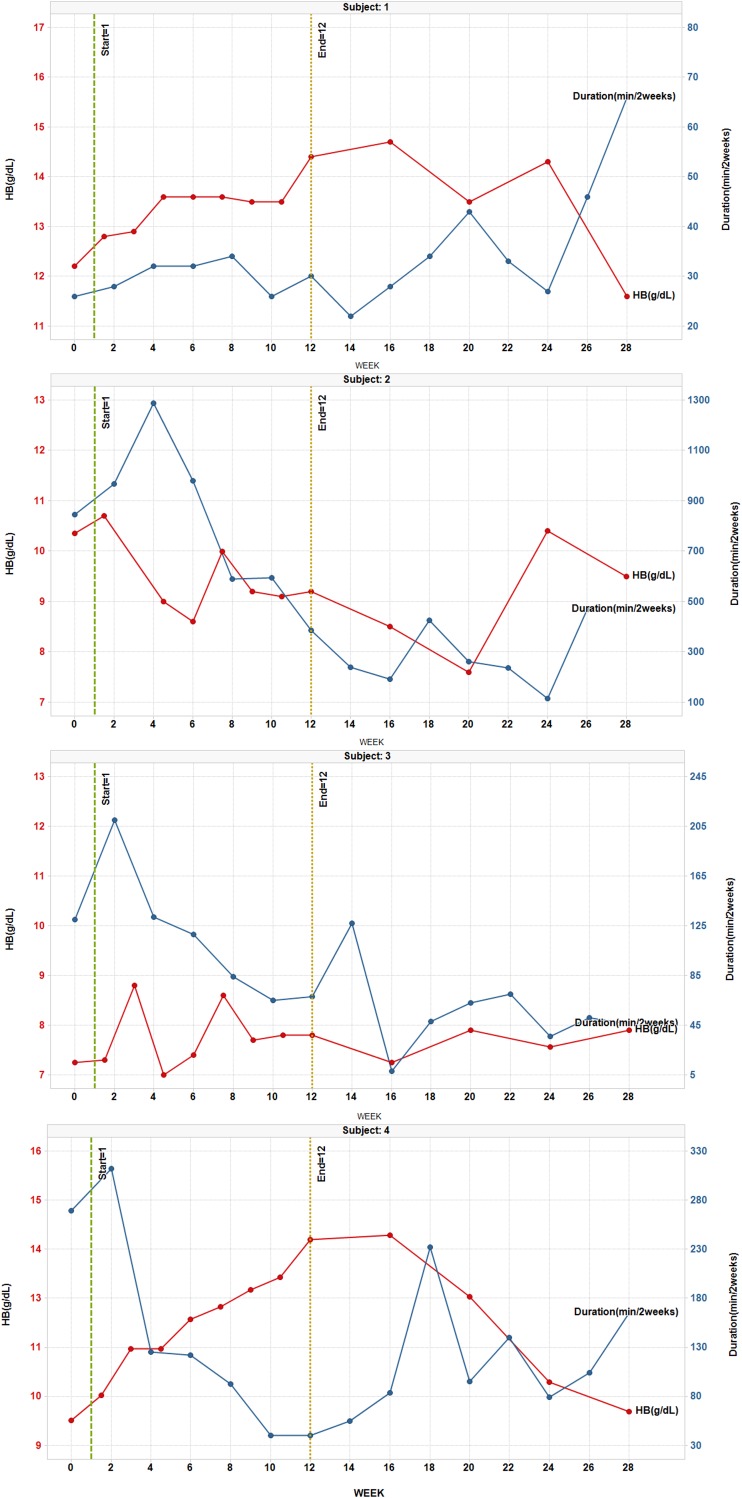

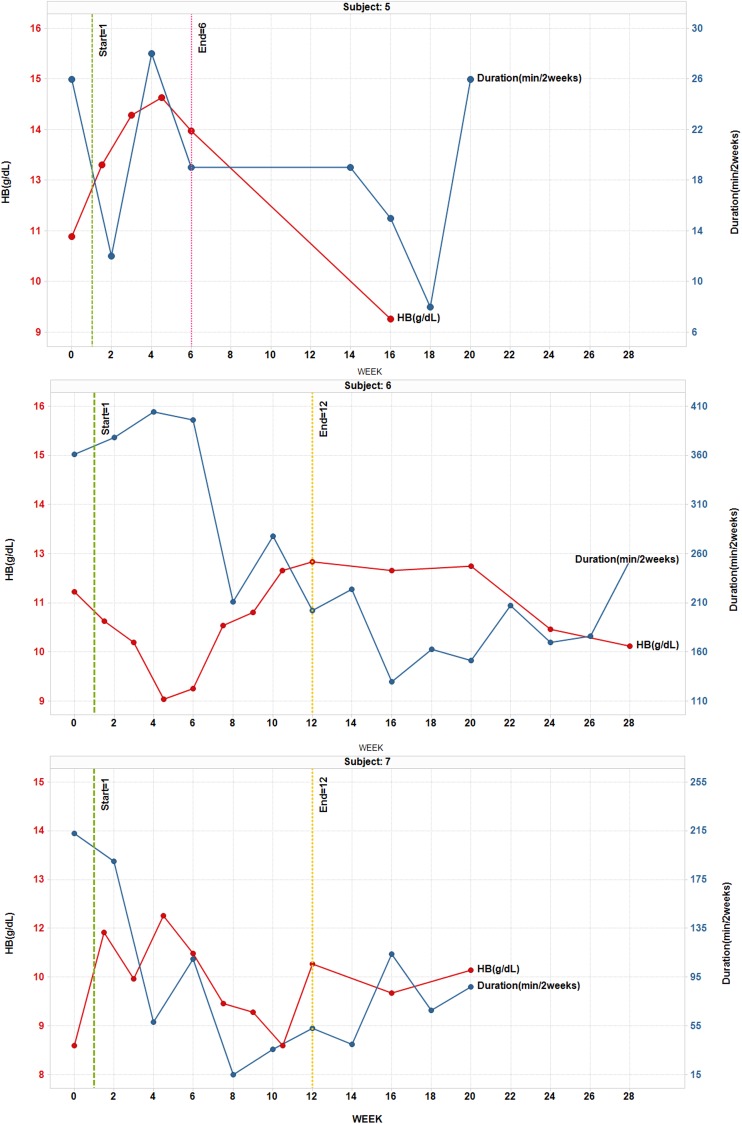
HB (gm/dL) and duration of epistaxis (min/2 weeks) for each patient, Reported at baseline, during therapy, and post-treatment follow-up

Six patients showed at least a 50% decrease in epistaxis duration relative to baseline at some point during the study and three of these showed at least a 50% decrease in duration during Weeks 11 and 12 (Table 1; Fig. 1). Six of six patients who reported gushing at baseline showed at least a 50% decrease in gushing relative to baseline at some point during the study and two of these showed at least a 50% decrease in gushing during Weeks 11 and 12. Five patients showed at least a 50% decrease in epistaxis frequency relative to baseline at some point during the study but two of these also showed at least a 50% increase in frequency at some point. Four patients were felt to have an overall improvement in epistaxis frequency (Table 1). Patient 4 showed an especially rapid and prolonged decrease in epistaxis duration, gushing, and frequency, which tracked with his improvement in Hgb (Fig. 1).

#### Hemoglobin, iron, and transfusion needs

Four patients showed more than a 2 gm improvement in Hgb relative to baseline at one or more points during the study and 2 (Patients 2 and 6) showed more than a 2 gm decrease at one point during the study (Table 1; Fig. 1). The only patient who had received RBC transfusion during baseline showed a marked decrease in RBC need during the study (none during weeks 4–18) and despite this decrease in RBC requirements did not show a drop in Hgb. Although Patient 5 was only on treatment for 6 weeks, he nonetheless showed a brisk increase in Hg during weeks 1.5–6, followed by a decline in Hg after stopping treatment. Patients 3, 4, and 6 showed a clear pattern of decreased iron infusion relative to baseline and the other four showed no overall change (Table 1). Patient 6 showed an early drop in Hg at week 4.5 which may have been related to stopping iron infusions, and then rebounded slightly above baseline at week 10.5. There was no appreciable change in ferritin levels for any patient during the study but the importance of this was difficult to assess due to variable iron infusion practices. Overall, all but Patient 2 showed one or more signs of hematologic response to treatment.

### Other outcome measures

**Fig. 2:**
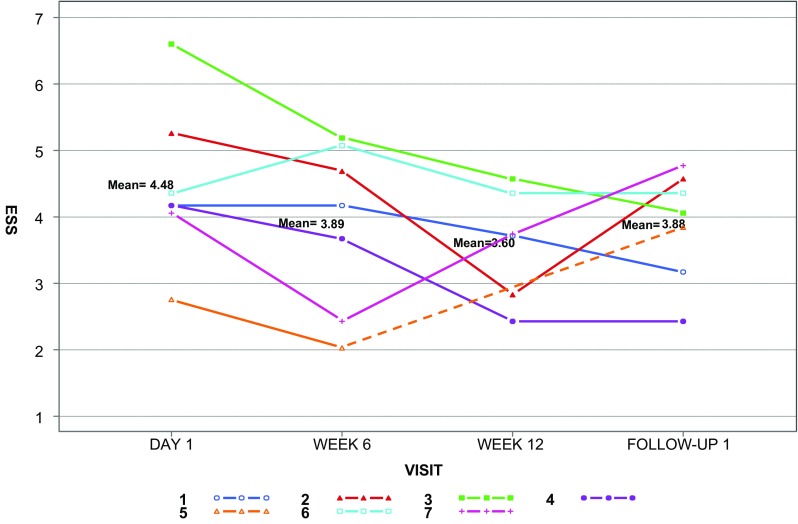
Epistaxis severity score (ESS) for each patient, with group means, reported at baseline, after 6 and 12 weeks of therapy, and at follow-up. The dashed line represents the time period after drug discontinuation in Patient 5

Six patients showed a decrease in ESS > 0.71 (the MID) relative to baseline at some point during the study and three of these showed a sustained improvement (Table 1; Fig. 2).

Most patients reported a generally positive experience during the semi-structured exit interviews, with six of seven patients indicating they were satisfied (*n* = 3) or very satisfied (*n* = 3) with the study treatment. Patient 4 was “very satisfied” and commented that “I’ve not experienced life without chronic anemia for many years and so it’s just a whole new world.” Patient 5 was the only one who was “neither satisfied nor dissatisfied” and this was mainly related to early discontinuation of study treatment due to liver function abnormalities.

Health-related QOL scores (norm-based) improved on all SF-36 domains (Table 3) except for general health, for which there was no change at Week 6 or Week 12. At Week 6, the MID was achieved or exceeded on six of the eight SF-36 domains. Scores on five of the eight domains exceeded the MID threshold at Week 12. Four domains showed improvement at both Weeks 6 and 12.

**Table 3:** SF-36 outcomes

	Day 1 (*n* = 7)	Week 6 (*n* = 7)	Week 12 (*n* = 6)	MID
Bodily pain	44.40 (8.3)	49.37^a^ (11.6)	42.58 (7.6)	3
General health	43.41 (12.6)	44.17 (11.7)	43.20 (7.7)	2
Mental health	43.56 (11.2)	47.81^a^ (10.7)	48.63^a^ (6.0)	3
Physical function	39.41 (10.3)	40.52 (9.4)	45.38^a^ (5.0)	3
Role emotional	38.74 (14.6)	48.01^a^ (10.8)	44.19^a^ (7.6)	4
Role physical	42.34 (10.6)	45.48^a^ (8.3)	45.38^a^ (9.1)	3
Social functioning	43.32 (10.2)	50.38^a^ (6.8)	41.91 (8.3)	3
Vitality	44.26 (7.9)	48.93^a^ (9.0)	46.47^a^ (5.1)	2

Values are mean ± standard deviation of norm-based scores. Day 1 and Week 6 are *n* = 7; Week 12 is *n* = 6 due to Patient 6 dropping out early

*MID* minimum important difference

^a^Indicates those time-points that improved greater than the MID versus Day 1

### Adverse events

Seven patients experienced a total of 19 adverse events (AE) (Supplementary Table). Two patients experienced bronchitis and two experienced nausea; all other AE were isolated occurrences. Four patients experienced a total of six suspected drug related AE including nausea (*n* = 2), abdominal distension (*n* = 1), dizziness (*n* = 1), headache (*n* = 1), and elevated alanine aminotransferase (*n* = 1). All AE were considered mild to moderate in intensity except for Patient 5 who had elevated alanine aminotransferase (considered severe). That patient experienced an elevation in elevated alanine aminotransferase to five times the ULN at Week 6 and was withdrawn from the dosing phase of the study. The alanine aminotransferase value was within normal limits at an unscheduled follow-up visit 21 days after discontinuing study drug. There were no serious AE. Additionally, no patient experienced a significant elevation in blood pressure or prolongation of QT interval.

### Pharmacokinetic data

Individual plasma pazopanib trough concentration values were obtained at Weeks 3, 6, 9, and 12 after once daily administration of 50 mg pazopanib (one patient only had two values).

Based on the elimination half-life of pazopanib (approximately 1 to 2 days), all patients were expected to be at steady-state by Week 3. High inter-patient variability was observed as average trough values ranged from 0.2 to 19.1 μg/mL (average weekly trough values ranged from 3.3 to 4.8 μg/mL). These trough values in HHT patients are generally similar to the trough values observed in patients with solid tumors who received 50 mg pazopanib once daily and had trough values obtained at approximately Weeks 1, 2, and 3 (individual range: 1.43 to 10.3 μg/mL; average weekly range: 3.77–4.66 μg/mL). Although there is high variability within some of the trough concentration profiles, patients typically appeared to exhibit steady-state conditions.

## Discussion

We report here the first human trial of pazopanib for treatment of HHT-related bleeding. In this small series, we observed an improvement in Hgb and/or epistaxis in all treated patients. This was observed at a dose much lower than typically used for oncologic indications, with no serious adverse events. These data suggest that further studies of pazopanib efficacy are warranted, as this drug may bring much-needed benefit to HHT patients suffering from chronic bleeding.

We studied patients who had chronic HHT-related bleeding, all suffering from epistaxis and secondary anemia, and six from GI bleeding. Study patients appeared similar to typical HHT patients with these types of complications, with a mean age in the fifties and the population divided equally between men and women, as well as *ENG* and *ACVRL1* gene mutations, with one patient with the rarer *SMAD4* gene involvement. As such, it is reasonable to consider these results as generalizable to other HHT patients.

We elected to measure hemoglobin (Hgb) as a primary outcome but to also study other measures (iron requirements, transfusion requirements, epistaxis measures, QOL and patient satisfaction) since the degree of anemia is itself dependent on supportive therapy. We agreed on pre-determined efficacy criteria. All but one patient had improvement in objective measures (Hgb and/or iron infusion requirements) and all had improvement in subjective measures. Specifically, four patients showed an improvement in Hgb which occurred despite no change or a decrease in iron and /or RBC infusion. In further support of drug effect, three of these four patients showed an obvious fall in Hgb 8–20 weeks after stopping pazopanib. An additional two patients showed stable Hgb despite reductions in iron and/or RBC infusion. A recent case report of an HHT patient treated with pazopanib 100 mg per day reported similar effect [29].

We hypothesized that pazopanib (which blocks VEGF receptors) would reduce bleeding based on preclinical data and anecdotal data derived from intravenous and topical bevacizumab use in HHT patients. Most of the bevacizumab evidence is associated with the standard 5–10 mg/kg oncologic dose which has shown substantial toxicity in cancer patients, as has the approved 800 mg once daily dose of pazopanib. However, we speculated that a lower dose of pazopanib would be safer and could still be effective given that it blocks VEGF-R2 signaling downstream of all of its ligands, including VEGF-A, and also affects other angiogenic signaling pathways including PDGF. In addition, we demonstrated in a mouse model of post-traumatic choroidal neovascularization that pazopanib activity is observable at a trough concentration of 1.3 µg/mL (approximating once daily human dosing of 15 mg) [30].

Benefit was observed across a range of total trough concentration values, including reduction in epistaxis duration in a patient with a low plasma pazopanib level of approximately 1 µg/mL and marked improvement of both hemoglobin and epistaxis duration in a patient with a high plasma pazopanib level of approximately 13 µg/mL. Due to the high degree of protein binding [> 99%] of pazopanib, the unbound active concentration is small, variable, and difficult to measure. An assumed protein binding level of 99.5–99.9% therefore results in a 3.98–19.9 ng/mL average free drug concentration at trough within our study which approximates the spectrum of IC50 biochemical and proliferative cell-based protein assays for VEGF and PDGF. These levels appear otherwise inadequate for tumor reduction, potentially due to the exuberant production of VEGF in tumors, and the restricted pharmacologic access of pazopanib to neoplastic tissue. Optimal oncologic levels are estimated at > 40 µM (i.e., >17.5 µg/mL) [31] providing perhaps as well a more complete and prolonged inhibition.

The exact mechanism by which pazopanib reduces bleeding is unclear. Involution of the tips of the growing vessel, resorption of the abnormal tuft of capillary and venule connections within an AVM, or general involution of abnormal vessels may be more likely than a reported direct hemodynamic effect [32]. This is consistent with the need for roughly 3 weeks of therapy prior to observing drug benefit, and then the loss of effect well beyond the expected systemic residence of the drug.

One patient [15%] revealed a substantial elevation in ALT, commonly reported [20–30%] in the oncologic literature. In contrast, the far more common elevation in blood pressure at high doses was not observed in our patients. In Phase I oncology studies, Pk values above 20 μg/mL resulted in substantive blood pressure elevation in > 50% of patients, compared to 18% in those with levels < 20 μg/mL [33]. Given the range of our trough concentration values, we might presume that the lack of virtually any BP rise was the result of IC50 versus IC90 levels of drug, or intrinsic HHT-related vascular protection from such influences. Overall, these much lower steady-state levels of pazopanib, while effective in HHT patients, appear generally devoid of these side effects.

The study is clearly limited by the small sample size and the absence of controls. The benefits across all seven patients (and all three typical HHT genotypes) are however provocative, particularly given the chronicity of the disease, and the baseline comprehensive care. All seven patients were already receiving expert care at specialized HHT Centers, receiving maximal medical therapy, and yet had ongoing anemia, chronic bleeding, and impaired QOL. In other words, pazopanib was added to the ongoing stable comprehensive care regimen in these patients, and we observed both subjective and objective benefit. To date, there have been few therapies in HHT with such promising preliminary data. Further study, with a controlled clinical trial, as initially planned, is now all the more relevant.

In conclusion, pazopanib offers a viable approach towards treatment of chronic bleeding in HHT patients. All seven patients in this study, at a dose of 50 mg daily for 12 weeks, realized a benefit, although of variable individual value, based on objective measures and patient reported outcomes. A far lower dose of drug is necessary to that of oncology indications. While safety remains unproven at this dose, and for this population, a window does appear to be available to effect benefit, without major safety risk.

## Electronic supplementary material

Below is the link to the electronic supplementary material.

Supplementary material 1 (PDF 72 KB)
