# Supplementary material for: Pazopanib may reduce bleeding in hereditary hemorrhagic telangiectasia
Source: Angiogenesis. 2018 Sep 6;22(1):145–55. doi: 10.1007/s10456-018-9646-1 (PMC6510884; doi:10.1007/s10456-018-9646-1)
Supplement: Supplementary file 1 — Supplementary material 1 (PDF 72 KB) [file 10456_2018_9646_MOESM1_ESM.pdf]

Supplementary Table of all adverse events. Seven patients experienced a total of 19 adverse events (AE) as detailed. All events were considered mild or moderate in severity, except one event (*) with elevation of alanine aminotransferase.

**System Organ Class**<br>**Preferred Term**	**GW786034: 50 mg QD**<br>**(N=7)**
Any Event, n (%)	7 (100)
Infections and infestations, n (%)	0
Any event	4 (57)
Bronchitis	2 (29)
Nasopharyngitis	1 (14)
Otitis media	1 (14)
Pharyngitis streptococcal	1 (14)
Musculoskeletal and connective tissue disorders	
Any event	4 (57)
Arthralgia	1 (14)
Muscle spasms	1 (14)
Musculoskeletal stiffness	1 (14)
Pain in extremity	1 (14)
Rheumatoid arthritis	1 (14)
Investigations	
Any event	3 (43)
Alanine aminotransferase increased*	1 (14)
Gamma-glutamyltransferase increased	1 (14)
Weight decreased	1 (14)
Gastrointestinal Disorders, n (%)	
Any Event	2 (29)
Abdominal distension	1 (14)
Nausea	2 (29)
Injury, poisoning and procedural complications	
Any event	2 (29)
Muscle strain	1 (14)
Sunburn	1 (14)
Nervous system disorders	
Any event	2 (29)
Dizziness	1 (14)
Headache	1 (14)
Psychiatric disorders	
Any event	1 (14)
Confusional state	1 (14)
